# Inpatient mortality in transition-aged youth with rheumatic disease: an analysis of the National Inpatient Sample

**DOI:** 10.1186/s12969-020-0416-4

**Published:** 2020-03-30

**Authors:** Paul T. Jensen, Keumseok Koh, Rebecca E. Cash, Stacy P. Ardoin, Ayaz Hyder

**Affiliations:** 1 Intermountain Healthcare, St. George, USA; 2 University of Hong Kong, Hong Kong, China; 3 Massachussetts General Hospital, Boston, USA; 4 The Ohio State University and Nationwide Children’s Hospital, Columbus, USA

**Keywords:** Transition to adult care, Pediatric rheumatology

## Abstract

**Background:**

Transition from pediatric to adult care is a vulnerable time for youth with chronic diseases. In youth with rheumatic disease, studies show high rates of loss to follow up and increased disease activity. However, mortality data are lacking. In this study, we assessed whether transitional age is a risk factor for inpatient mortality.

**Methods:**

We analyzed the 2012–2014 National Inpatient Sample database, a representative sample of discharges in the United States. Individuals with rheumatic diseases were identified by International Statistical Classification of Disease – 9 (ICD-9) codes at time of discharge. Youth were categorized into three age groups: pre-transitional (11–17), transitional (18–24) and post transitional (25–31). We fitted univariable and multivariable logistic regression models to assess whether transitional age was a risk factor for inpatient mortality.

**Results:**

There were 30,269 hospital discharges which met our inclusion criteria of diagnosis and age. There were 195 inpatient deaths (0.7%). The most common causes of death were infection (39.5%), pulmonary disease (13.8%), and cardiac disease (11.2%). The Odds ratio for inpatient mortality of a transitional-aged individual was 1.18 compared to controls (*p* = 0.3). Black race (OR = 1.4), male sex (OR = 1.75), and a diagnosis of systemic sclerosis (OR = 4.81) or vasculitis (OR = 2.85) were the greatest risk factors of inpatient mortality.

**Conclusion:**

Transitional age was not a risk factor for inpatient mortality in this study. We did identify other risk factors other than age. Further studies are required to assess if there is an increased risk of mortality in outpatients of the transitional age group.

## Background

Each year in the United States, approximately 500,000 adolescents with chronic health problems turn 18 and become young adults [1]. Young adulthood can be a vulnerable time, especially for individuals with chronic medical conditions [2]. The period of transfer from pediatric to adult care has been associated with many adverse health outcomes including graft failure among kidney transplant recipients [3, 4], increased risk of hospitalization, [5] worse glycemic control in patients with diabetes [6] and loss of viral suppression in patients with HIV [7]. Some studies have even raised concern for increased mortality during and shortly after the transfer period in liver transplant recipients [8] and youth with HIV [9].

For these reasons there has been increased attention given to transition, the comprehensive processes of “purposeful, planned movement of adolescents and young adults with physical and medical conditions from child-centered to adult oriented health systems [10].” Transition is much more than just the transfer from a pediatric provider to an adult provider. Rather it encompasses all aspects of the patient’s health as he/she takes more responsibility for his/her own condition [11]. Pediatric healthcare is frequently paternalistic and family oriented with little responsibility assigned to the patient while adult healthcare values patient autonomy and responsibility. Additionally, the emergence of adulthood ushers in additional stressors such as changes in insurance status, education, housing, and employment. Many organizations including the American Academy of Pediatrics, American College of Physicians, and American Academy of Family Physicians have written guidelines to improve the process of transition [1, 12] and many “transition programs” have been developed to improve adolescent and young adult health have been developed with differing results [2, 13, 14].

A majority of youth with pediatric onset rheumatic conditions have persistently active disease into adulthood [15–19]. The need therefore exists to prepare these youth for adult healthcare systems and transfer them in a way that minimizes adverse effects on health and wellbeing [20]. For this reason, the European League against Rheumatism (EULAR) released rheumatology specific guidelines for the transitioning of youth. Many pediatric rheumatology clinics have developed rheumatology specific transition programs [21–25].

However, the data on adverse outcomes related to suboptimal transition are not as robust in rheumatology as they are in other fields. Studies on clinical outcomes are limited to small retrospective series [26] or use loss to follow up as a surrogate for poor transition [27]. To date, there are no studies assessing whether there is an increase in mortality during the transition period in youth with rheumatic disease. In this study, we used data from the National Inpatient Sample (NIS), a representative database of inpatient hospitalizations in the United States [28], to determine whether transition aged youth with rheumatic disease have increased inpatient mortality compared to similar patients who were not of transition age.

## Methods

The NIS is a representative sample of 20% of hospital discharges in the United States. The data are collected from “all non-Federal, short-term, general, and other specialty hospitals” with some exclusions including correctional facilities" [29] and is estimated to be a representative sample of 95% of hospitals. The data set was redesigned in 2012 by the Agency for Healthcare Quality and Research (ARHQ). One significant change made was the state where hospitalization occurred was removed. This made census division (a grouping of several states) the smallest geographic unit by which discharges could be analyzed [30].

We combined the 2012, 2013, and 2014 NIS into a single data set as these three years used the same methodology. Inclusion criteria for this study were patients with rheumatic disease, as identified by ICD9 code, and age 11–31 years at discharge. We excluded records with incomplete or erroneous ICD9 codes and records in which it was not evident whether the patient was alive or dead at the time of discharge.

The following ICD9 codes were used to identify patients with rheumatic diseases: systemic lupus erythematosus (710.0), systemic sclerosis (710.1), Sjogren’s syndrome (710.2), dermatomyositis (710.3), polymyositis (710.4), eosinophilia myalgia syndrome (710.5) mixed connective tissue disease (710.8), undifferentiated connective tissue disease (710.9), rheumatoid arthritis (714.0–714.2), juvenile idiopathic arthritis (714.3), ankylosing spondylitis (720.0), seronegative inflammatory spondyloarthropathies (720.2–720.9), psoriatic arthritis (696.0), other inflammatory arthritis (714.8–714.9), periodic fever syndrome (277.31), Henoch-Schonlein purpura (287.0), polyarteritis nodosa (446.0), Kawasaki disease (446.1), hypersensitivity angiitis (446.2), ANCA associated vasculitis (446.3–446.4), giant cell arteritis (446.5), Takayasu’s arteritis (446.7). The patient was considered to have a rheumatic disease if one of these diagnoses appeared in the first 30 diagnoses listed at time of discharge.

Due to NIS requirements, data cells with fewer than 10 outcomes were combined to protect subject identity. The following combinations were created: inflammatory arthritis (rheumatoid arthritis, juvenile idiopathic arthritis, ankylosing spondylitis, inflammatory spondyloarthropathy, and other inflammatory arthritis); other connective tissue disease (dermatomyositis, polymyositis, eosinophilic myalgia syndrome, mixed connective tissue disease, and undifferentiated connective tissue disease); vasculitis (Henoch Schonlein purpura, ANCA associated vasculitis, polyarteritis nodosa, Kawasaki Disease, hypersensitivity angiitis, giant cell arteritis, and Takaysu’s arteritis). If a record contained a diagnosis in more than one of these categories, it was counted separately in each category where it had a fitting diagnosis.

The first listed ICD-9 code for patients who died during hospitalization was considered to be the cause of death. Once again due to NIS requirements regarding cell size, the causes of death were combined into the following categories: infectious diseases (e.g. sepsis, cellulitis, tubercular disease, and medical device infection), cardiac diseases (e.g. pericardial disease, myocardial infarction, arrhythmias, and heart failure), neurologic disease (e.g. seizures, coma, cerebral hemorrhage and cerebral thrombosis), trauma or poisoning (e.g. skull or vertebral fractures, drug overdose, and traumatic vessel injuries), pulmonary disease (e.g. non-infectious pneumonia, respiratory failure, and pulmonary emboli), and miscellaneous conditions (e.g. unspecified hemorrhage, intestinal problems, malignancy or other unrelated comorbidities.) Although all patients had an autoimmune disease as part of the entry criteria, the autoimmune disease was considered to be the primary cause of death if it was listed as the first discharge diagnosis.

Individual records were categorized by age at discharge as being pre-transitional (defined as ages 11–17 years), transitional aged (18–24) or post-transitional (25–31). Texas does not report age at discharge to the NIS, but instead reports mid-points of age ranges; the age ranges are different for patients with sensitive conditions and non-sensitive conditions. Sensitive conditions are defined by the State of Texas as HIV, alcohol abuse, and drug abuse. We used the age reported as actual age; the age reported was compatible with our ranges for patients with non-sensitive conditions [31].

### Statistical analyses

Each covariate of interest (age grouping, sex, income quartile of zip code, race, and disease) were entered into univariable logistic regression models to estimate odds of death while in the hospital. The complex sampling techniques of the NIS were addressed in analysis. Discharges with missing data fields were excluded from the specific analyses in which those data fields were needed. Discharges with missing mortality data (*n* = 3) were excluded completely from analyses. No imputation was performed. Three sequential multivariable logistic regression models were then created to estimate the odds of death based on demographic information, hospital-specific information, and specific rheumatic disease.

All were then entered into a multiple logistic regression model. Transitional age status was given special status and forced into the multivariable model without regards to its significance. The multivariable logistic regression model was then improved by reverse selection. The covariate with the highest *p*-value by Wald statistic was removed and then the model was tested against the previous model by the likelihood ratio test. The covariate was removed from the model if the p-value by likelihood ratio test was > 0.05. The covariate with the highest remaining p-value was then tested in a similar fashion. On removal of each covariate, analysis for confounding was done by assessing the effect on remaining covariates.

After removal of all covariates which were not significant by the likelihood ratio test, the remaining covariates were then tested for interaction with other covariates individually with *p* = 0.01 being used as the cutoff for interaction terms remaining in the model. Test of goodness of fit was then performed using the Hosmer-Lemeshow tests and discriminate function using the area under the receiver operating curve tests. All statistical tests were done using STATA 14.0 (StataCorp LLC, College Station, Texas).

This study was approved by the Institutional Review Board of Nationwide Children’s Hospital (IRB18–01277) and was carried out in accordance with a Data Use Agreement from the Agency for Healthcare Research and Quality (ARHQ). All data were de-identified on receipt from AHRQ and no attempt was made to re-identify individuals.

## Results

There were 21,488,293 individual hospital discharges in the combined NIS data sets from 2012 to 2014. Of those 3,163,380 (14.7%) were between the ages of 11 and 31. Of the remaining observations, 30,269 (0.96% of all admissions in the age group) were found to have a rheumatic disease by ICD9 coding as described. The patients tended to be overwhelmingly female, and towards the older end of the allowed age range. The demographic information of the patients with rheumatic diseases can be found in Table 1. More than one rheumatic diagnosis was noted in 2879 (9.5%) records with the highest number being patients who were coded as having both rheumatoid arthritis and systemic lupus erythematosus (*n* = 1124, 3.7%).

**Table 1:** Demographic information of the discharges which met the inclusion criteria from 2012 to 2014 NIS

	Unweighted n	Weighted % (95% CI)
Sex		
Female	24,726	81.7 (81.2–82.2)
Male	5540	18.3 (17.8–18.8)
Age		
11–17	3557	9.6 (8.8–10.4)
18–24	9743	26.3 (25.8–26.9)
25–31	16,966	64.1 (63.2–65.0)
Race		
White	12,320	40.7 (39.8–41.6)
Black	8986	29.7 (28.8–30.6)
Hispanic	4993	16.5 (15.7–17.3)
Other	2272	7.5 (7.1–7.9)
Missing/Unknown	1695	5.6 (5.0–6.3)
Economic Status		
Top 25%	5429	18.2 (17.5–19.1)
50–75%	6713	22.6 (22.0–23.2)
25–50%	7555	25.4 (24.8–26.1)
Bottom 25%	10,006	33.7 (32.8–34.6)
Private insurance	10,968	29.6 (29.0–30.3)

One hundred and ninety-five hospitalizations (0.7%) ended in patient death while 30,074 ended in the patient being alive. Patient status at time of discharge was not available for 3 individuals who were excluded from further analysis. The most common primary diagnoses (and likely causes of death) of individuals who died were infection (*n* = 77, 39.5%), pulmonary disease (*n* = 27, 13.8%), and cardiac disease (n-22, 11.2%). Furthermore 21 patients (10.8%) had their autoimmune disease listed as primary discharge diagnosis. Primary diagnoses of individuals who died during hospitalization can be found in Table 2.

**Table 2:** Primary diagnoses for individuals who died/Presumed cause of death from the 2012–2014 NIS

Primary Diagnosis	n	Percentage
Infection	77	39.5
Pulmonary Disease	27	13.8
Cardiac Disease	22	11.2
Primary Autoimmune Disease	21	10.8
Trauma/Overdose	15	7.7
Neurologic or Cerebrovascular Disease	13	6.7
Miscellaneous	21	10.2

Of the qualifying rheumatic diagnoses, those with systemic sclerosis and vasculitis diagnoses died at much higher rates compared with other rheumatic diagnoses. Rheumatic diagnoses and associated death rates can be found in Table 3. Other significant risk factors for inpatient mortality in the univariable models were male sex and black race. Having private insurance and having an elective admission were protective.

**Table 3:** Disease specific information and death rates

	With Disease		Died		
	Unweighted n	Weighted % (95% CI)	Unweighted n	Weighted n (SE)	Weighted % (95% CI)
Lupus	17,032	56.3 (55.5–57.0)	102	170.0 (16.9)	0.6 (0.5–0.7)
Scleroderma	604	2.0 (1.8–2.2)	15	25.0 (6.9)	2.5 (1.5–4.2)
Other connective tissue diseases	2476	8.2 (7.8–8.5)	14	23.3 (6.2)	0.6 (0.3–1.0)
Inflammatory arthritis	9464	33.2 (32.6–33.9)	44	75.0 (11.16)	0.4 (0.3–0.6)
Periodic fever syndrome	82	0.3 (0.2–0.3)	0	0	
Vasculitis	2502	8.3 (7.9–8.7)	41	68.3	1.6 (1.2–2.3)

**Fig. 1:**
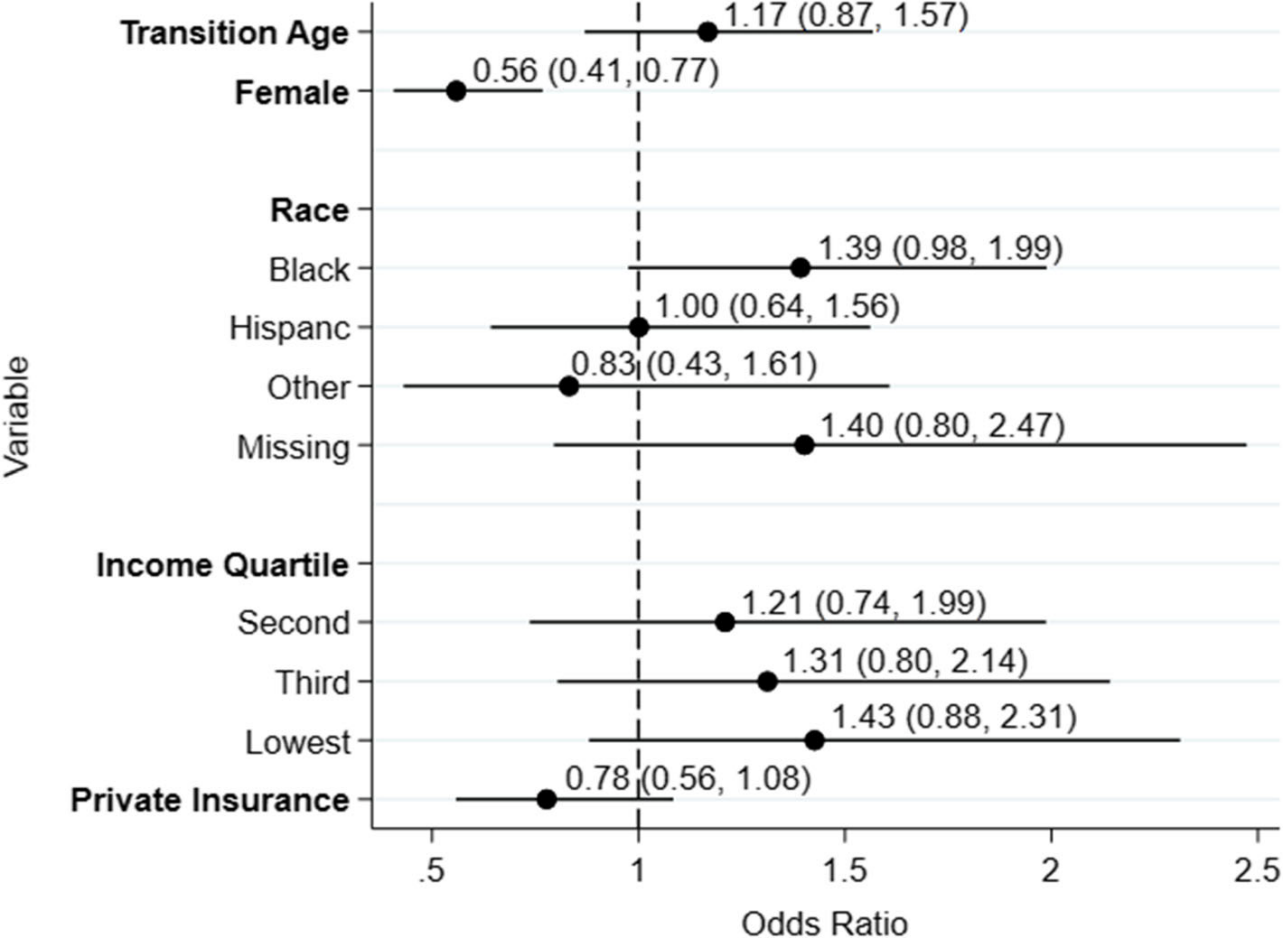
Odds ratios for death in the demographic-only multivariable model. White race and highest income quartile are the referent classifications for race and income quartiles respectively. The dashed line represents an odds ratio of 1.0

**Fig. 2:**
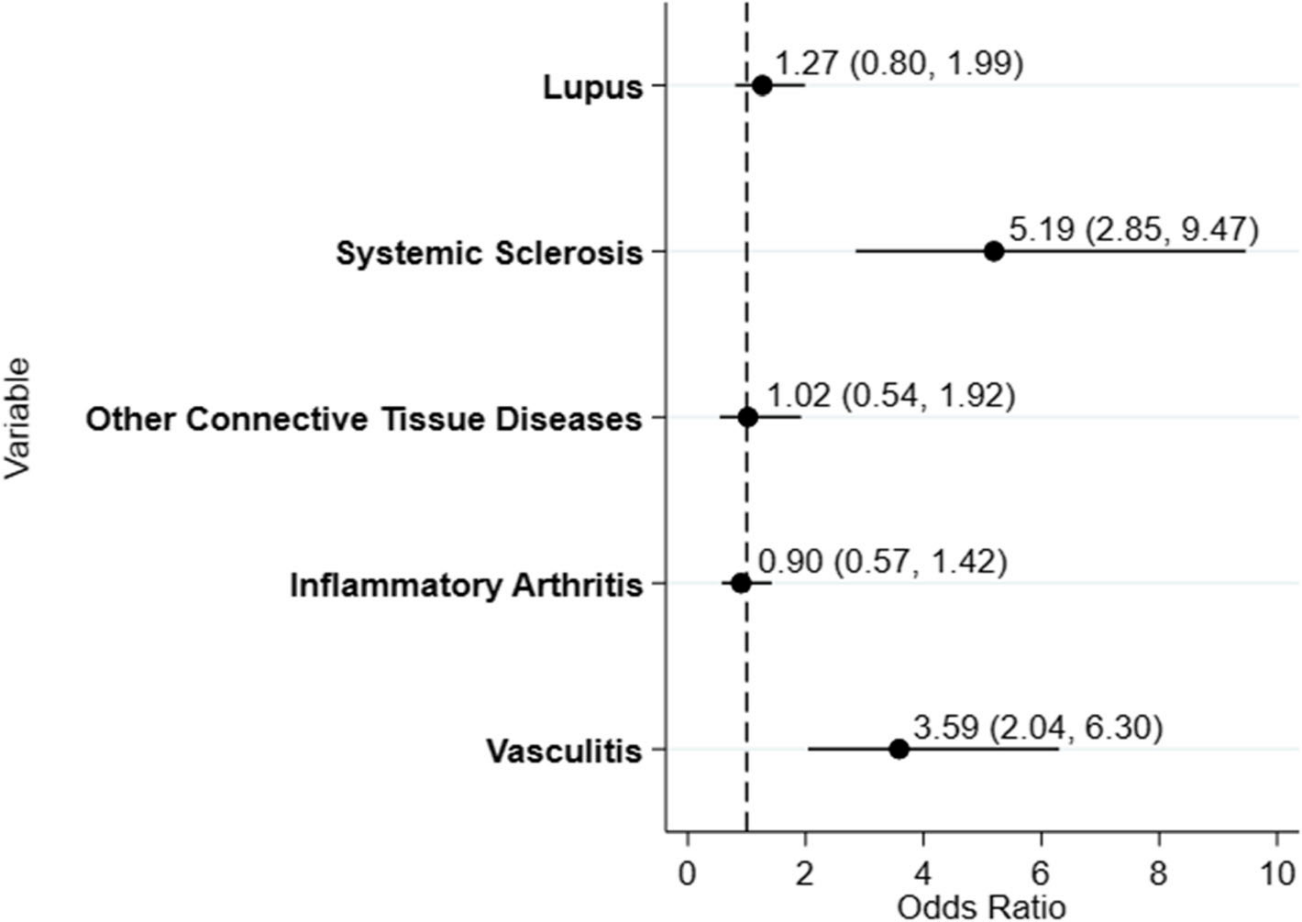
Odds ratios for inpatient mortality based on Disease Model The dashed line represents an odds ratio of 1.0

In the demographic-only multivariable model (Fig. 1), sex was the only significant risk factor for death. The demographic only model had an area under the ROC curve of 0.61 and a Hosmer-Lemeshow χ^2^ statistic of p-0.87. The disease specific model (Fig. 2)0 had an area under the ROC curve of 0.62 with a Hosmer-Lemeshow χ^2^ statistic of p-0.97.

**Fig. 3:**
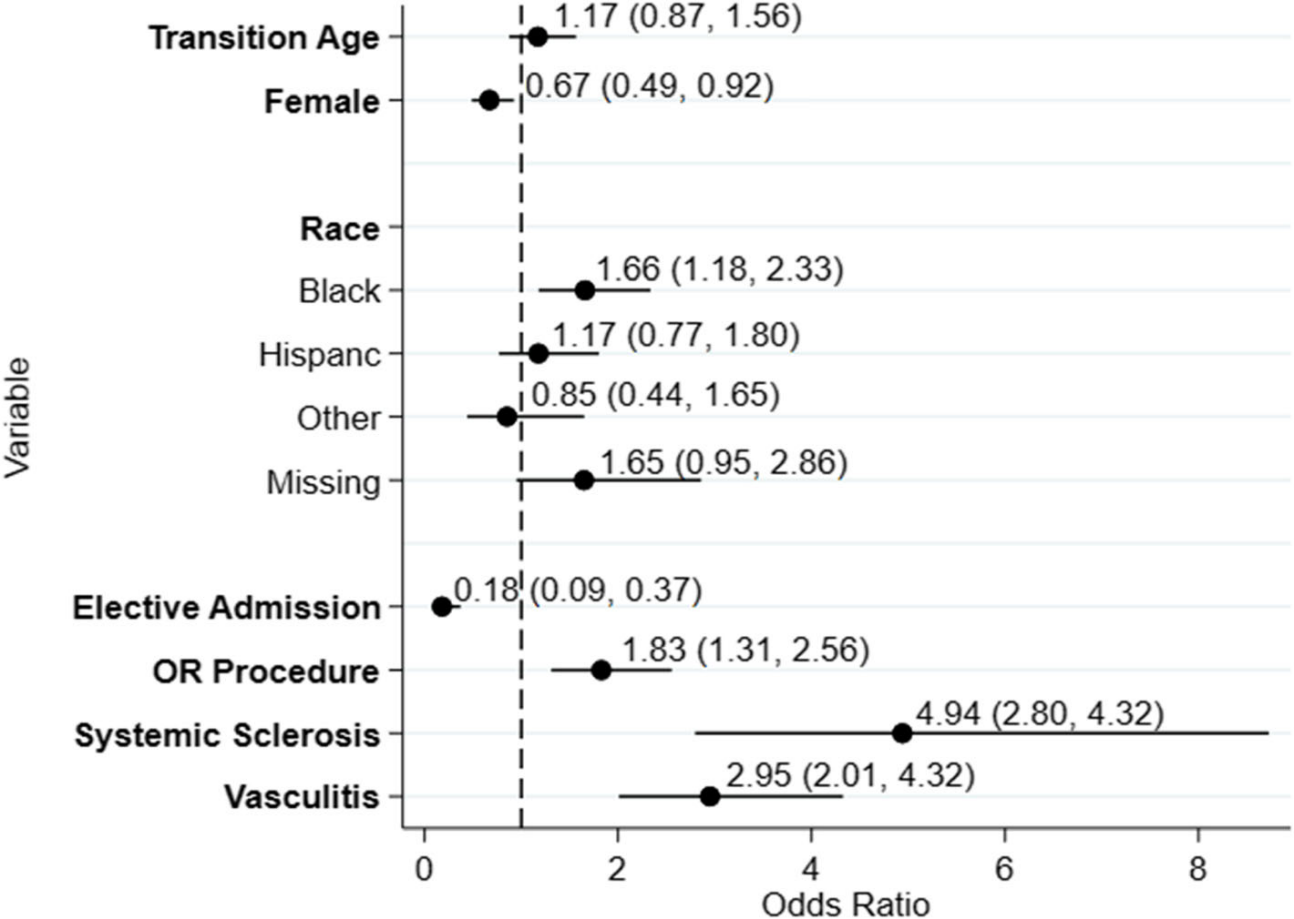
Odds ratios for inpatient mortality based on the combined multivariable logistic regression model The dashed line represents an odds ratio of 1.0

The final model included female sex, race, whether the admission was elective, and whether there was an operating room procedure as well as the diagnoses of scleroderma or vasculitis were significant predictors of death. (Fig. 3) This model had no evidence of lack of fit by Hosmer-Lemeshow goodness of fit test (*p* = 0.75). The area under the receiver operating curve was 0.67.

## Discussion

This study is, to our knowledge, the largest assessment of mortality in transitional aged youth of any disease. Previous studies on the outcomes of transitional aged youth with rheumatic diseases have either focused on loss to follow up as a surrogate for clinical outcomes [24, 27] or have been small single site studies [26]. We have shown for the first time that being a youth of transitional age was not associated with increased inpatient mortality in youth with rheumatic diseases, though similar findings were found in a Japanese cohort of patients with type 1 diabetes [32]. This study represents the first time that the National Inpatient Sample has been used to assess transition and it is the largest study of transition outcomes in a rheumatic disease population.

Although our primary hypothesis of increased mortality in transition aged youth was not significant, we did identify several at-risk groups who may benefit from increased research as well as increased support during the transition process. Black race and male sex were associated with increased odds of inpatient mortality in youth with rheumatic disease. The cause of increased mortality in these groups is not clear given the relatively small number of deaths in any one group. However, a recent report from the Centers for Disease Control and Prevention found increased mortality among Blacks compared to other racial groups for all age ranges under 65 though this gap had narrowed recently [33] and a study by Yen et al. showed that decreases in lupus mortality over the last 46 years were slower in Blacks individuals compared to White individuals [34]. Among adolescents with cancer, an increase in mortality rates among African Americans was found to be mainly associated with insurance status and socioeconomic status [35] which also seemed to be at play in our study though it was not significant and not included in the final model. While the odds of inpatient mortality in Black race individuals improved when controlling for insurance status and income quartile of ZIP code, increased odds of death did still persist. This suggests that additional non-economic factors are also contributing to the increased odds of death. African American individuals with lupus were found to have more comorbidities than white individuals with lupus in a recent retrospective cohort, especially hypertension [36] We believe that transition programs focusing on both economic factors and control of comorbidities may help reduce the racial gap in youth of transition age. Further study into the specific features of transition aged youth including cause of death for each age and racial group is required.

This work also documented presumed causes of death of hospitalized adolescents and young adults with rheumatic diseases. Although use of a first discharge diagnosis as a presumptive cause of death may be problematic, we assume that the primary diagnosis would be a key contributor to cause of death. Our finding that 39% of youth who died had infectious diseases as their primary diagnosis demonstrates once again that infection is a major complication of the rheumatic diseases. The rate of infection we found was higher than reported by Butt et al. in a Danish cohort with systemic sclerosis [37] and by Aggarwal et al. in an Indian cohort of youth with lupus [38] but similar to the Chinese lupus cohort of all ages by Mu et al. [39]

We also found that patients with vasculitis and scleroderma were at higher risk of dying in the hospital relative to patients with other rheumatic diseases (of which lupus was the most frequent comparator diagnosis). While this can be explained by the increased severity of these diseases, they are understudied in the transition literature [40, 41]. Our findings of increased mortality suggest that perhaps vasculitis and systemic sclerosis should receive special attention during the transition process as well as more research.

Despite the use of a large database, our study had several limitations. We used transition-aged youth as a surrogate for youth who are actually transferring from pediatric to adult care. While the use of the NIS database made it impossible to assess transition directly, the use of age as a surrogate risks bias due to the “ecologic fallacy” in which youth noted to be of transitional age may not have indeed being those who were transferring care [42]. Additionally there is the risk of misclassification of age. As noted previously, Texas does not report the true age of patients with “sensitive” conditions. Those with a history of HIV, alcohol abuse, or drug abuse aged 1–17 are reported as being age 8 and would not have been in our data set. Those aged 18–44 are reported as being 31 and therefore some who were of transitional age may have been misclassified as being “post-transition [31].” There is also a possibility of misclassification of patients due to incorrect ICD-9 coding. Several studies and reviews have noted poor identification of patients with rheumatic diseases such as SLE [43, 44], rheumatoid arthritis [45], dermatomyositis [46], and ANCA associated vasculitis [47]. Unfortunately techniques to improve the specificity of case detection used in other administrative databases such as use of an ICD code at two separate events, the incorporation of specific laboratory values (e.g. ANA, rheumatoid factor,) the incorporation of immunomodulatory medications, or having a code noted by a rheumatologist were not possible due to the setup of the NIS data set.

Finally, the data were collected only from inpatients. A plausible explanation of our non-significant primary outcome would be that young adults of transitional age are more likely to die prior to being admitted to the hospital, either in the emergency department setting or at home without seeking out medical services. A future study that includes data from both inpatient and outpatient services or with a prospective cohort would be necessary to understand non-inpatient deaths among transitional aged youth. The JIA and SLE registries being created by the Childhood Arthritis and Rheumatology Research Alliance (CARRA) may provide an opportunity to better answer these questions.

## Conclusion

Our use of the NIS data set has provided the first assessment of the effects of transition age on mortality. Although the effects of transition age were not statistically significant, we did identify risk factors for death in a young patient population including male sex, non-white race, and diagnoses of vasculitis or scleroderma. These factors can serve as triggers for more intensive medical or socioeconomic interventions to improve outcomes and save lives. More research is needed into clinical outcomes (including death) in transition aged youth who are not admitted to the hospital.

## Data Availability

The National Inpatient Sample 2012–2014 data sets are available for purchase from the US Agency for Healthcare Research and Quality at https://www.hcup-us.ahrq.gov/db/nation/nis/nisdbdocumentation.jsp

## Abbreviations

AHRQ: United States Agency for Healthcare Research and Quality
ANA: Anti Nuclear Antibody
ANCA: Anti Neutrophil Cytoplasmic Antibody
EULAR: European League Against Rheumatism
HIV: Human Immunodeficiency Virus
ICD: International Statistical Classification of Disease
NIS: National Inpatient Sample
OR: Odd’s Ratio
